# Fidelity of end joining in mammalian episomes and the impact of Metnase on joint processing

**DOI:** 10.1186/1471-2199-15-6

**Published:** 2014-03-22

**Authors:** Abhijit Rath, Robert Hromas, Arrigo De Benedetti

**Affiliations:** 1 Department of Biochemistry and Molecular Biology, Louisiana State University Health Sciences Center, 1501 Kings Highway, Shreveport, LA 71130, USA; 2 Department of Medicine, College of Medicine, University of Florida & Shands, Gainesville, FL 32610-0277, USA

**Keywords:** Accuracy of DSB repair in mammalian cells, Episomal model of NHEJ, End- processing and re-ligation, Metnase nuclease, Joint accuracy

## Abstract

**Background:**

Double Stranded Breaks (DSBs) are the most serious form of DNA damage and are repaired via homologous recombination repair (HRR) or non-homologous end joining (NHEJ). NHEJ predominates in mammalian cells at most stages of the cell cycle, and it is viewed as ‘error-prone’, although this notion has not been sufficiently challenged due to shortcomings of many current systems. Multi-copy episomes provide a large pool of genetic material where repair can be studied, as repaired plasmids can be back-cloned into bacteria and characterized for sequence alterations. Here, we used EBV-based episomes carrying 3 resistance marker genes in repair studies where a single DSB is generated with virally-encoded HO endonuclease cleaving rapidly at high efficiency for a brief time post-infection. We employed PCR and Southern blot to follow the kinetics of repair and formation of processing intermediates, and replica plating to screen for plasmids with altered joints resulting in loss of chloramphenicol resistance. Further, we employed this system to study the role of Metnase. Metnase is only found in humans and primates and is a key component of the NHEJ pathway, but its function is not fully characterized in intact cells.

**Results:**

We found that repair of episomes by end-joining was highly accurate in 293 T cells that lack Metnase. Less than 10% of the rescued plasmids showed deletions. Instead, HEK293 cells (that do express Metnase) or 293 T transfected with Metnase revealed a large number of rescued plasmids with altered repaired joint, typically in the form of large deletions. Moreover, quantitative PCR and Southern blotting revealed less accurately repaired plasmids in Metnase expressing cells.

**Conclusions:**

Our careful re-examination of fidelity of NHEJ repair in mammalian cells carrying a 3′ cohesive overhang at the ends revealed that the repair is efficient and highly accurate, and predominant over HRR. However, the background of the cells is important in establishing accuracy; with human cells perhaps surprisingly much more prone to generate deletions at the repaired junctions, if/when Metnase is abundantly expressed.

## Background

Double Stranded Breaks (DSBs), a serious type of chromosome damage are usually caused by exogenous agents such as ionizing radiation (IR), topoisomerase poisons, radiomimetic drugs like bleomycin and doxorubicin, or endogenous damages which arise as a result of cellular metabolism (free radicals and DNA replication errors or collapse of stalled forks). Programmed DSBs are also generated during processes such as V(D)J recombination of immunoglobulin genes and during allelic exchanges at meiosis or zygotic fusion. If misrepaired, DSBs can result in chromosomal translocations, oncogenesis, and genomic instability [1-3]. DSB repair can be broadly divided into two major pathways: homologous recombination (HR) and non-homologous end joining (NHEJ) [4]. HR requires the presence of an intact homologous template, often a sister chromatid, which allows accurate repair of DSBs via a replicative intermediate and is the preferred mode of repair in S and G2 phase of cell-cycle [5]. In contrast, NHEJ operates without the need for a template DNA; sometimes joining the ends with minimal processing, hence considered potentially erroneous/mutagenic. NHEJ is the predominant form of repair in mammalian system and had been shown to operate all throughout the cell cycle [6].

Currently used DSB repair assays have several shortcomings. Researchers employ several methods such as Ionizing radiation (IR), radiomimetic drugs, laser, and site-specific endonucleases for induction of DNA damage [7]. Of note, γ-irradiation and drug induced damage foci are randomly dispersed and non-homogenous in nature. Lasers create localized but exceedingly strong DNA damage. In addition, different wavelengths of the laser largely determine the damaged end structures subsequently resulting in widely different kinetics of repair with differential protein requirement (for a review see [7,8]). DSBs introduced with a nuclease are adequate for studies of the molecular/cellular mechanisms, but estimates of repair accuracy are problematic since these enzymes turn over slowly and thus selecting for erroneous processing of the ends that eliminate the target sequence. This is well-known for the Endonuclease I-SceI induced DSBs [9], a model that also suffers from the need of pre-integration of the target sequence, and often low cutting efficiency (either due to low transfection of the cell population or because of the chromosomal location of its target sequence). The Endonuclease I-Ppol also suffers from low cleavage efficiency because of its unique target location in the highly condensed and repetitive ribosomal DNA gene clusters. Furthermore, study of repair at defined genomic DSBs is problematic due to the difficulty in re-obtaining ‘clean clonal’ isolates for analysis in sufficient representation. Likewise, use of yeast as a model system to parlay mammalian NHEJ is not entirely acceptable. Yeast like *Saccharomyces cerevisiae* or *Saccharomyces pombe* lack many well established components of mammalian NHEJ, like DNA-PKcs and Artemis. To date, orthologs for newly discovered NHEJ components of human APLF, PNK, Metnase, and APTX have not been identified in yeast [10]. Furthermore, HR is the preferred repair pathway in yeast, whereas NHEJ is the predominant pathway in mammalian cells [11]. In yeast, IR-induced and endonuclease-induced DSBs are differentially processed in a cell cycle-dependent manner [12].

Hence, we set out to develop one model system to efficiently represent repair activities in mammalian cells in a physiological setting, with episomes. Repair of multi-copy plasmids provides a large pool of mostly uniform genetic material where the repair process can be studied [13-15]. We utilized the yeast HO endonuclease (a key component of the mechanism for mating-type switch in yeast) for use in mammalian cells. In this system, the HO endonuclease is expressed by a recombinant adenovirus resulting in cleavage of its target site (on episomes) with high efficiency (depending on the MOI), and repair occurs via simple end-joining during a time course of infection. We previously utilized a mouse mammary cell line containing a single integrated HO target site at a defined genomic location [16]. Whereas, this has generated powerful and useful information, for example in terms of the effects of chromatin on generation of the DSB and in repair [16,17], for other purposes the system is limiting. For instance, damage induced by IR or genotoxins results in multiple simultaneous DSBs instead of a single break, which immediately raises questions in terms of similarity of activation and deactivation of the DNA damage response (DDR). It is of significant interest to the field of mammalian DSB repair to develop a model system, where there is synchronous induction of multiple ‘homogeneous’ site-specific DSBs in a population, and then be able to stop the nuclease activity rapidly [18].

Different assays, such as treatment of pre-cleaved plasmids with cellular extracts or transfection of linearized DNA templates into mammalian cells have been employed to study several different aspects of end-joining (efficiency of joining of different DNA ends, fidelity of repair [13,19-21]). However, technical limitations prevent a clear picture from emerging regarding the end-joining efficiency/fidelity and nature of sequence rearrangements. For instance, *Smith et al.*, using a transfection assay reported a similar efficiency of end-joining for different DNA ends. However, *Poplawski et al.*, using cellular extracts have reported widely varying degree of efficiency of end-joining with different end structures [13,22]. In fact, a recent report presented evidence for strikingly different repair efficiency using either lipid-based transfection vs. nucleofection [23]. This highlights the importance of conducting end-joining assays in a ‘nearly unperturbed’ cellular environment. Where looked at repair fidelity of a DSB at an integrated chromosomal locus [24] most studies were limited in terms of low cleavage efficiency, absence of multiple defined site-specific DSB sites, and also the persistent activity of the nuclease employed. We thus wanted to investigate the repair fidelity in an episomal population. It is well established that EBV-based episomes become chromatinized and behave as minichromosomes [15,25]. High density MNase maps for EBV-based episomes have been generated that have revealed highly positioned nucleosomes and putative origins of replication [26]. Hence, repair studies of minichromosomes can also bring into focus the contribution of chromatin, closely resembling genomic damage. Finally, we wanted to characterize the effect of a recently identified important component of human NHEJ, known as Metnase, as an endonuclease on episomal repair fidelity in intact cells. Metnase has been shown to be a general facilitator of NHEJ increasing both accurate and inaccurate repair [27]. Mutation of its nuclease domain has been shown to prevent this role, at least in a subset of end-joining events [28,29]. Using *in vitro* assays, Metnase has been shown to have a preference for single stranded DNA overhang of a partially duplex molecule with an effect more pronounced on a 3′ overhang [29]. Evidence from experiments done with plasmid-host cell transfection system, point to a significant role of Metnase in determining repaired junction fidelity for breaks with 3′ overhangs. Also, in a transfection assay coupled with integration, over-expression of Metnase did not significantly increase accurate NHEJ at the break site [27]. *Beck et al.*, reported a role of Metnase in promoting end-joining with non-compatible ends (both 5′ & 3′) in a cell free system [29]. However, a very recent study reported against any functional role of Metnase in promoting end-joining of modified DNA ends using a similar cell free system [30]. These contradictory findings hint towards a gap in knowledge regarding the role of Metnase as an endonuclease processing different ends [31], and even greater in intact cells. We wanted to determine the contribution of Metnase in altering repair fidelity (accurate vs. inaccurate repair) in our model system and to characterize the nature of nucleotide rearrangements to understand more in depth NHEJ fidelity in human cells.

## Methods

### Preparation of stable cell lines maintaining episomal HO-CAT plasmid

The 34 bp HO endonuclease target sequence (agatcttttagtttcagctttccgcaacagtata) was cloned in to the HindIII site of the pREP4/CAT shuttle vector just before the chloramphenicol acetyl transferase (CAT) coding sequence and the plasmid was renamed as HO-CAT plasmid. Further, HO-CAT plasmid was transfected in to 293 T cells which maintain the plasmid episomal due to presence of EBNA-1, under hygromycin selection (Hygromycin B/Calbiochem, Cat # 400052). The cell line was named 293 T-HO-CAT. Similarly HEK293 cells were transfected and selected to produce the 3-HO-CAT cell line. Metnase expressing pCAPP-Metnase-V5 plasmid which was prepared in Robert Hromas’ lab was transfected in to 293 T cells and puromycin (Puromycin Dihydrochloride/MP biomedicals, Cat # 100552) selected to produce the Metnase over expressing 293 T stable cell line. Subsequently, HO-CAT plasmid was transfected into these cells to produce the Met-T-HO-CAT cell line and maintained under dual selection of hygromycin and puromycin. GenePORTER (Genlantis, Cat # T201007) was used as the transfection reagent for all the above mentioned transfections. All the three cell lines were cultured using DMEM with 10% FCS as growth medium at 5% CO2 and 37°C.

### Assay for DNA cleavage and repair

In a typical assay, for each different time point, 150,000 cells were infected with the recombinant adeno virus encoding HO endonuclease (Gift from Dr. Hamish Young, Columbia University, NY, USA) at 10–30 MOI, referred to as pPF446::HO in a plasmid map in [32]. DNA from cells was collected using either Wizard SV genomic DNA purification system (Promega, Cat # A2360) or Wizard plus SV miniprep DNA purification (Promega,Cat # A1460) protocol. Equivalent amount of DNA was used in PCR and qPCR reactions for CAT and AMP regions using GoTaq DNA polymerase kit (Promega) and DynAmo Flash SYBR Green qPCR kit (Thermo Scientific, Cat # F-415S) respectively. 5′-CTACAACAAGGCAAGGCTTGACC-3′ and 5′-TCTAGTTGTGGTTTGTCCAAACTCATC-3′ were used as forward and reverse primers respectively for CAT amplicon. 5′-TTCCGTGTCGCCCTTATTCCC-3′ and 5′-GGCACCTATCTCAGCGATCTG-3′ were used as forward and reverse primers respectively for AMP region in PCR reactions. PCR conditions: 30 cycles of 94°C for 30 s, 55°C for 30 s, and 72°C for 39 s with a final extension of 10 minutes at 72°C. The CAT signal was normalized to AMP signal. For qPCR, 5′-GTACCAGCTGCTAGCAAGCT-3′ and 5′-TCAACGGTGGTATATCCAGTGAT-3′ were used as forward and reverse primers respectively for the CAT region (amplicon size 133 bp). 5′-CATCGAACTGGATCTCAACAGCG-3′ and 5′-GTCATGCCATCCGTAAGATGCT-3′ were used as forward and reverse primers respectively for AMP region.

### Immunoblotting

Protein samples from different time points were collected by RIPA lysis buffer (50 mM Tris pH 8.0, 0.1% SDS, 1% Triton-X, 1 mM EDTA, 150 mM NaCl, and with 1X protease inhibitors – SIGMAFAST protease inhibitor cocktail tablets, Cat # S8820) and quantitated using BCA protein assay kit (Pierce, Cat # 23223). Equal amounts were run on a 12% polyacrylamide gel, blotted on to Immobilon-P (Millipore, Cat # IPVH08100), and subsequently probed with anti-E3-11.6 K antibody (fusion protein with HO). The antibody was a kind gift from Dr. William S.M. Wold, St. Louis school of medicine, Missouri, USA. The blot was re-probed with Phospho- (S/T)Q-ATM/ATR substrate antibody (Cell Signaling, Cat # 2909S). Anti-Rabbit-HRP conjugated antibody (Cell signaling, cat # 7074S) was used as the secondary antibody. Stripping was done using Re-Blot plus mild (Millipore, Cat # 2502). The blot was either developed using Pierce ECL reagent (Thermo Scientific, Cat # 32106) or Opti-4CN (Bio-Rad, Cat # 170–8235).

### NheI or NotI screening and bacterial transformation

Plasmids recovered from each different time point were subjected to NheI (or NotI) digestion (Promega, Cat # R6501) which has its recognition sequence very close to HO induced cleavage site. Subsequently, the enzyme was heat inactivated and the miniprep DNA was used to transform XL-1 Blue supercompetent cells (Agilent, Cat # 200236) and plated on an Ampicillin plate (100 μg/mL).

### Replica plating assay

Plasmid DNA isolated from different cell lines at different time points was used to transform was incubated for 16 h at 37°C and subsequently replica plated on a chloramphenicol plate (50 μg/mL). Colonies were counted with Bio-Rad ChemiDoc (Cat # 170–8265) machine using Quantity-One software.

### Luciferase assay

The HO-Luc plasmid was transfected using GenePORTER (Genlantis, Cat # T201007) and concomitantly infected with adeno-HO virus in either 293 T-HO-CAT cells or 293 T cells. At each time point, cells were taken of the plate and one aliquot of it was used to extract the DNA to be used for PCR to assay cleavage and repair. The rest of the cells were assayed for luminescence using Luciferase Assay system (Promega, Cat # E1501). GFP fluorescence was used to normalize the luminescence values. Both luminescence and fluorescence were measured by Synergy 4 plate reader by BioTEK instruments.

### Southern Blotting for plasmid DNA

One fully confluent T75 flask was used for each time point. Plasmids were recovered using Zyppy plasmid Kit (Zymo Research, Cat # D4019). 10ug of plasmid DNA from each time point was digested with either Csp45I and NotI or Csp45I alone. Heat inactivated samples were run in 1% agarose-gel. Blotting onto Immobilon-Ny + (Millipore, Cat # INYC00010) was achieved using Trans-Blot SD semi-dry transfer cell (Biorad, Cat # 170–3957). EKONO hybridization buffer (Research Products International, Cat # 248800) was used for both pre-hybridization and hybridization. Hybridization probe was synthesized by random priming using random hexamers (Invitrogen, Cat # 51709) using whole plasmid DNA as template. Finally, the blot was exposed to X-ray films and developed using standard developer.

### Preparation of cell extracts

About 1 million cells were used to prepare the whole cell extract for each mentioned time point. Cells were collected off the plate and washed once with cold 1X PBS. Subsequently, the cells are washed once with hypotonic buffer (25 mM Tris pH 7.9, 1 mM MgCl2, 0.4 mM CaCl2, and 0.5 mM DTT), spun down, and resuspended again in 200 μL of hypotonic buffer and kept in ice for 20 minutes. The cells were homogenized in a tight fitting Dounce homogenizer (30 strokes) and the debris was spun down by spinning at 13000 rpm for 10 minutes at 4°C. The supernatant is collected and used in the DNA cleavage reaction.

### *In vitro* DNA cleavage assay

The reaction was carried out in a 10 μL reaction mixture with 250 ng of pMat-Puro plasmid, 6 mM MgCl2, 50 mM NaCl, 5 units of XmnI enzyme (Promega, Cat# R727A) (0.5 μL), and 2 μL cell extract for each given time. The mixture was incubated at 30°C for 1 h. Then, the reactions were stopped by adding 5 μL of stop solution (100 mM EDTA, 1% SDS, 1 mg/mL Proteinase K) and 3 μL of 6× loading dye and incubated at 55°C for 30 min-1 h. Then, the mixture was run in a 1% agarose gel.

## Results

### Development of an episomal model system to study DSB repair

**Figure 1:**
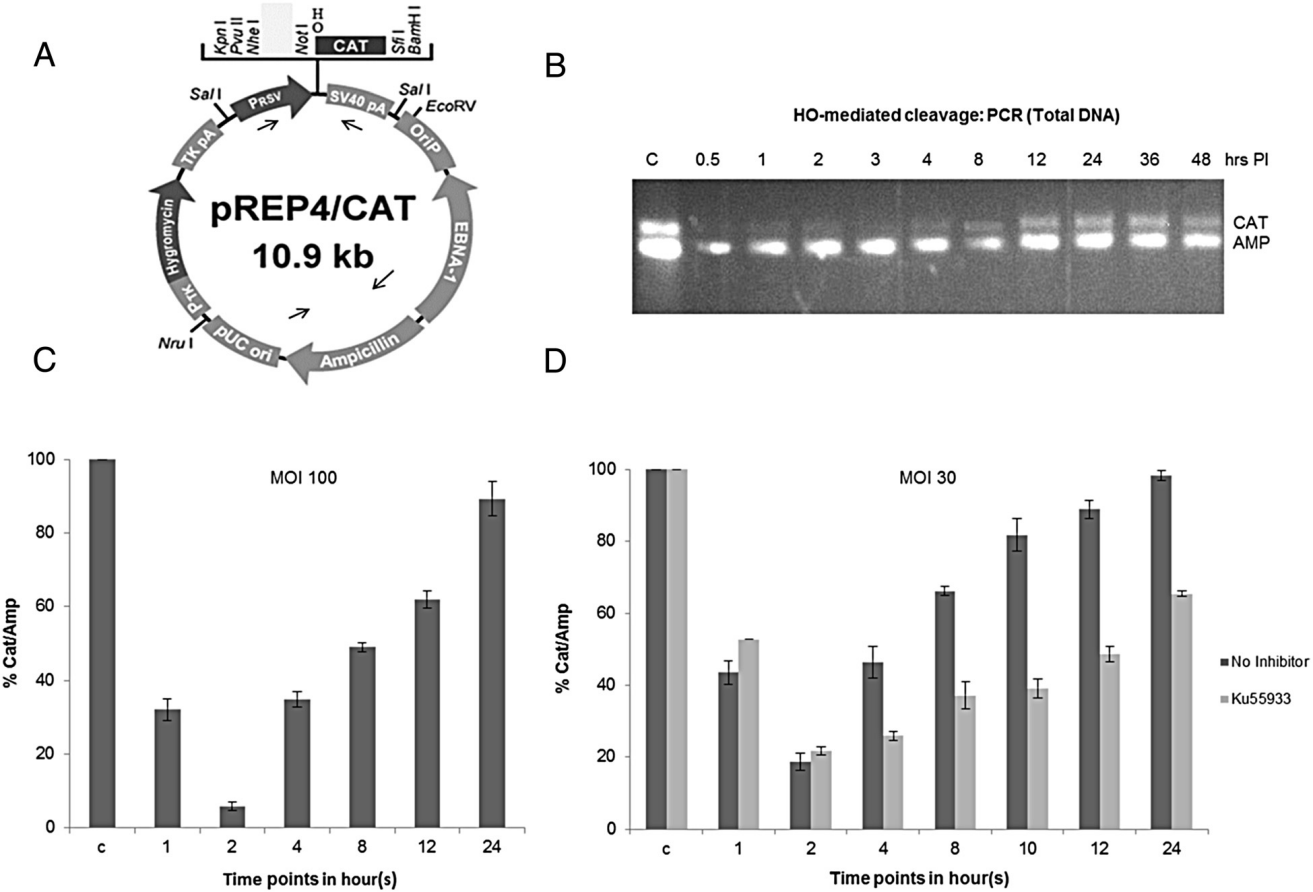
**Typical episomal cleavage and repair assay. (A)** Map of the pRep4-HO-CAT plasmid showing key features. HO recognition cassette is cloned in just before CAT gene. Arrows indicate location of the primers. **(B)** Episomal cleavage-and-repair assay at the HO site DSB during a time course of Ad-HO infection. T-HO-CAT cells were infected with adeno-HO (Ad-HO) virus, cells were collected at different time points, and episomes were recovered. PCR was performed by putting primers across the break site (CAT amplicon). AMP (Ampicillin) region on the episome acts as the positive internal control and used for normalization. In addition, a genomic product was also used to ensure equal total DNA recovery from the samples. **(C)** qPCR analysis for kinetics of cleavage and repair of epsiomes as explained in **(B)**. qPCR values are represented as (CAT/AMP)% over control. **(D)** Same as in **(C)** but in presence of the ATM inhibitior-KU55933 (10 μM).

We initially established a cell line (human 293 T) transfected with a shuttle vector with the HO target site cloned into it (293 T-HO-CAT) (see methods for detailed description). The vector is maintained as a stable copy number episomal unit inside the mammalian cells because of the presence of EBNA-1 protein encoded by the vector (Figure 1A) [33]. This provides us with multiple copies of homogenous template which become chromatinized and have been shown to accurately reflect repair of genomic DNA [14]. Minichromosomes are physiologically relevant but simple substrates to study DNA repair. Being homogenous in nature and also the ability to isolate them from bulk chromatin are some other advantages of the episomal system. We are conducting studies by sucrose gradient sedimentation of isolated episomes that have revealed highly ordered, chromatinized plasmid species (to be published elsewhere).

In a typical cleavage and repair assay, a single site specific DSB was generated in the episomal population by infecting the cells with recombinant adeno-virus (Ad-HO) encoded HO endonuclease [16,32] that recognizes a 34 base pair (bp) specific sequence and produces a DSB with a 4 bp 3′ overhang. Repair was followed in a time course after isolating the population of plasmids and using endpoint PCR and q-PCR detecting an amplicon spanning the break site (Figure 1B, CAT amplicon, Figure 1C). Upon induction of the DSB, the PCR product is lost, but then recovers with time as the repair proceeds. The Ampicillin region on the vector was used as an internal normalization control for plasmid recovery. The cleavage efficiency of HO-site was consistently >90% as judged from multiple experiments at 10–30 MOI. We have previously shown robust DDR activation due to HO induced site-specific cleavage across the episomal population that matches the kinetics of repair in terms of appearance and disappearance of S1981 phosphorylation of ATM (Figure 2 in [34]; reproduced with permission in Additional file 1: Figure S1). Such data confirm previous observations of DDR activation with as little as 10–20 DSBs or even with a single DSB [35-37]. To ensure that the episomal repair derives a significant contribution from the DDR, we treated the cells with the ATM inhibitor KU55933. The kinetics of repair in the drug treated cells was slower in comparison to untreated control (Figure 1D). These results show that in this system an active DDR pathway is elicited due to presence of multiple DSBs. Of interest, some previous studies have suggested the non-essential role of ATM during much of NHEJ events, based largely on evidence from mutant cell lines, except for instances of condensed heterochromatic sites [38]. In this episomal system, while we have abundant direct evidence that the plasmids are chromatinized and supercoiled, presence of heterochromatic regions are not believed to be likely. Since, for example, histone H1 is found only in small amounts in association with episomes (and their nucleosomes) after isolation on sucrose gradients [[25] and data not shown].

**Figure 2:**
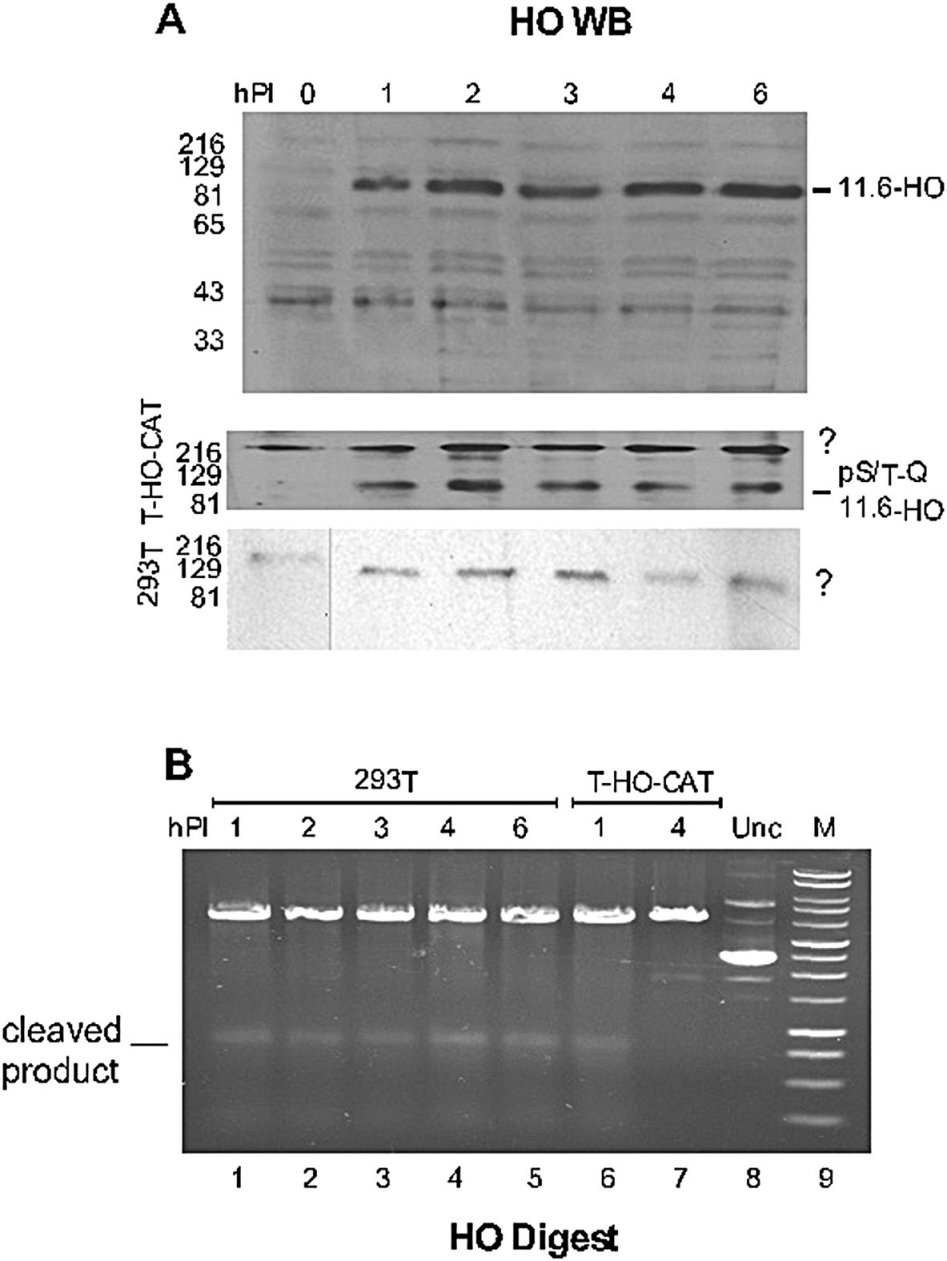
**Expression and activity of HO-fusion protein in permissive cell system (293 T cells). (A)** Depicts immunoblot of HO fusion protein upon infection with adeno-HO virus at different time points in 293 T-HO-CAT cells. A map of the virus showing the HO fusion is presented in [32], and generates a presumed chimeric protein with the 11.6 k adenovirus gene product. Cells were lysed using RIPA lysis buffer and equal amounts of proteins were loaded and run in a 10% SDS-PAGE gel and subsequently probed with anti-E3-11.6 K (fusion protein with HO) antibody. The blot in panel **(A)** was re-probed with phospho-(S/T) Q-ATM/ATR substrate antibody, and the region of the blot corresponding to the position of HO in panel (A) is shown. Also shown, the p(S/T)-Q banding pattern in 293 T cells which lack the episomes after Ad-HO infection, to serve as a control. ? denotes undetermined bands obtained after probing with p(S/T)-Q antibody. **(B)** HO activity was measured in an *in vitro* cleavage reaction. Cell extracts were prepared from 293 T cells (lanes 1–5) and cells carrying pRep4-HO-CAT episomes (T-HO-CAT cells) (lanes 6–7) after Ad-HO infection at given time points. The plasmid pMat-Puro, harboring the HO recognition site [16], was added exogenously at 250 ng per reaction. The plasmid was treated with cell extracts obtained from different time points from both 293 T cells and T-HO-CAT cells. HO activity was judged by presence/absence of a cleaved product. In reactions shown in lanes 1–7, we have added Xmn1 to produce the ~750 bp band and corroborate the cleavage at the HO site. Lane 8 shows the treatment of the pMat-Puro plasmid with extracts obtained from uninfected cells (Unc). “M” denotes the Promega 1 Kb DNA ladder as a molecular weight marker.

### Kinetics of HO expression and inactivation

In Figure 2 we show a typical experiment with the kinetics of HO expression. In initial studies, we have observed a second cleavage cycle between 24-36 h Post-infection (PI) that could be explained by release of new virus and reactivation of the promoter driving HO expression in 293 T cells. For a study of repair fidelity, it is important to have the cleaving endonuclease transiently active. This reduces the bias toward inaccurate repair of the joint introduced in the system by the recurrent cutting and accurate restoration of the recognition sequence. In this context, *Kaplun et al.*, have shown that HO is a target of Mec1/ATR mediated phosphorylation leading to its subsequent ubiquitination and degradation in yeast [39]; an obvious mechanism for haploid yeast to avoid repeated mating-type switching until the next cell division. We have previously shown that in a non-permissive cell system, HO expression shuts down by 3 h of infection, ensuring (mostly) a single round of cleavage/repair implicating the existence of a similar mechanism in mouse cells [40]. In these cells (MM3MG mouse cells - non-permissive to human adeno virus) the HO protein, is expressed soon after infection leading to cleavage of its target site. But subsequently, ATM activation, and phosphorylation of HO results in its proteolytic loss with no new net synthesis [16], thus explaining absence of recurrent cutting of the restored target site and the enzyme is then degraded.

However, in a replication permissive system (human 293 T cells) HO nuclease persists throughout the infection (Figure 2A) due to a complex pattern of activation of early and late promoters usage [41] driving the E3/11.6-HO fusion [42]. We should caution that the identification of this band, clearly a viral product, as the E3/11.6-HO fusion remains uncertain. Unfortunately a published HO antiserum is not available, and the way to detect the HO was through its fusion with the 11.6 k protein [32,42], but previously presence of this product had been correlated with the nuclease activity [16]. Assuming we have correctly identified the HO protein, however, it was unclear whether the HO enzyme remained active after cutting its target(s) or if it became nonetheless inactivated via its ATM/ATR-mediated phosphorylation. Maintenance of HO activity would elicit multiple rounds of cleavage and repair of the episomal targets, and the fundamental question was whether the pattern of repair (recovery of the CAT PCR product) was the result of a single round of cutting and repair, or a more complex pattern of cycles of cleavage and re-ligation. To answer this, first we determined that HO indeed became phosphorylated (by ATM/ATR) in this system, after we re-probed the HO-WB with a phospho-Ser/Thr (S/T) ATM/ATR substrate antibody (S/T-Q motif is the well characterized target of ATM/ATR substrates [43] and is also present in HO). Figure 2A shows rapid S/T-Q phosphorylation of the band co-migrating with HO that paralleled the cleavage of plasmid and the kinetics of ATM activation in this system [34] (Additional file 1: Figure S1). Treatment with λ phosphatase abolished the signal (not shown). In contrast, infection of plain 293 T cells showed a number of phospho-S/T-Q immune-reactive bands, but none that corresponded to the HO position or that corresponded to phospho-proteins induced after doxorubicin treatment (Additional file 2: Figure S2). In short, a possible scenario is that HO becomes S/T-Q phosphorylated after (maximal) cleavage of the bulk of episomes; and no longer seems to cleave until viral replication is complete and the next cycle of infection begins (the cells do not lyse until ~5-7 days later).

To confirm that HO is indeed inhibited following a single round of cleavage and repair, we performed two types of experiments, one with extract of HO-infected cells supplemented with a reporter plasmid exogenously and one in intact cells. We already knew by WBs that the HO fusion protein varies little throughout the time course of infection, but its activity seems to be limited to the first hour, after which the enzyme is phosphorylated by ATM/ATR and appears to be inhibited. We postulated that the source of ATM/ATR activation is the HO induced cleavage of HO-CAT episomes as shown previously (Figure 2 in [34]; Additional file 1: Figure S1). Hence, preparing the cell extract from Ad-HO infected 293 T cells not carrying the episomes would effectively avoid ATM/ATR activation, thus maintaining an active HO endonuclease. We thus prepared cell extract from both 293 T cells and cells carrying the episomes (T-HO-CAT cells) at different time points post infection with Ad-HO virus. As shown in Figure 2B, addition of pMat-puro plasmid (carrying a HO recognition site, [16]) to uninfected cell extract (lane 8) results in no appreciable cleavage (after 1 h of incubation *in vitro*). However, incubation in Ad-HO infected 293 T cell extracts collected at different time points results in complete linearization of the plasmid even after the first hour PI, and the HO activity remains so for the next 6 h, as judged by the appearance of cleaved product (we also added Xmn1 to these reactions to enhance the distinction between the linearized plasmid and the mobility of the relaxed circular form). In contrast, in cells carrying the pRep4-HO-CAT plasmid (labeled T-HO-CAT), the enzyme is active only for the first hour PI, but not after 4 h (note presence/absence of the cleaved product) (Figure 2B), indicating that the HO is inactive following cleavage of the endogenous episomes. Treatment of this ‘HO extract’ with λ phosphatase gave some return of cleavage but with unclear results as there was more plasmid smear (not shown).

**Figure 3:**
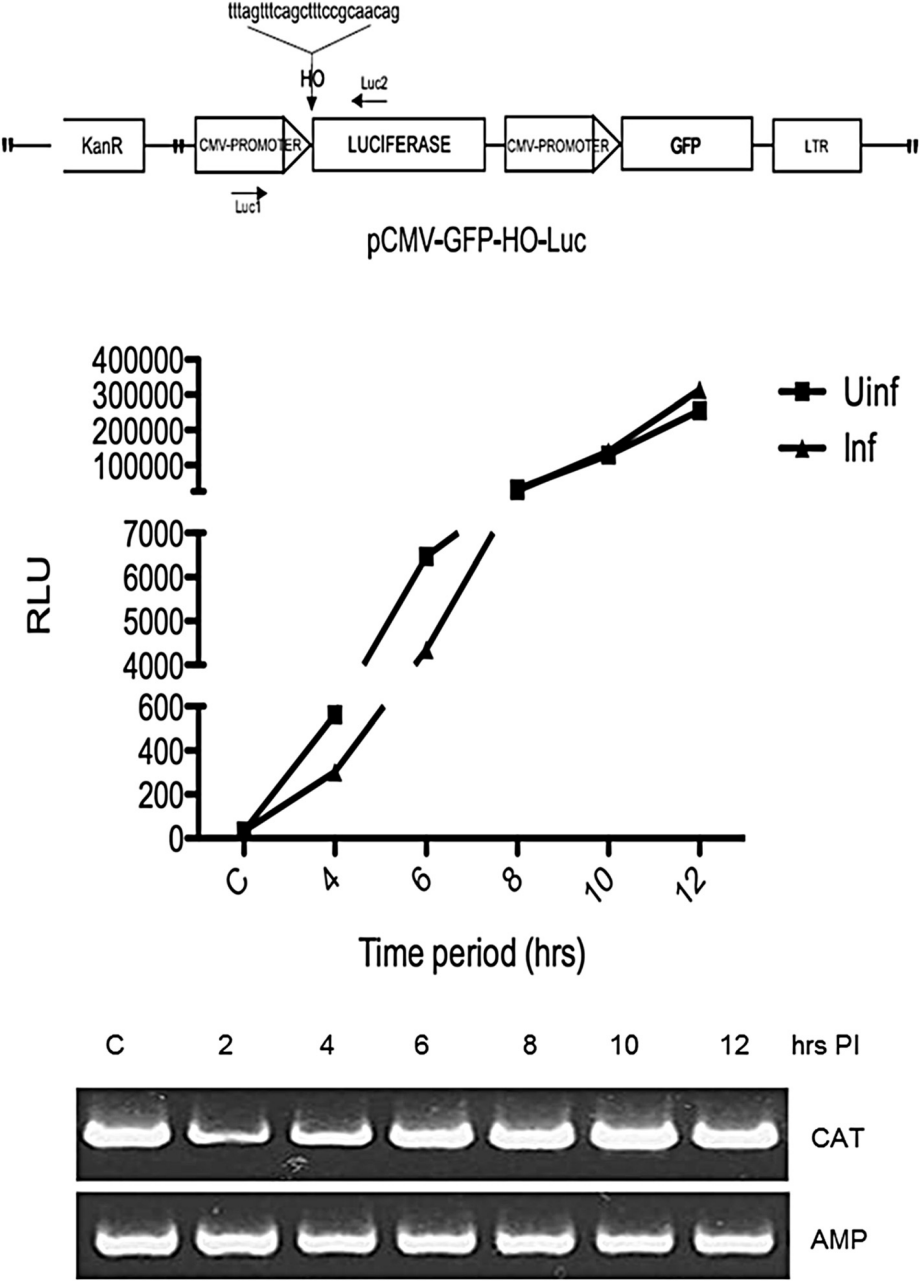
**Luciferase expression in 293 T-HO-CAT cells at different time points.** The cells were transfected with the vector construct with HO target site cloned in just before the Luciferase ORF (shown on top). Luciferase expression was measured in both uninfected and Ad-HO infected cells and normalized to GFP expression. Both infection and plasmid reporter transfection were carried out concomitantly. PCR shows the cleavage and repair kinetics across the time points in the HO-CAT episomes.

For the experiment *in vivo*, we transfected a Luciferase/GFP reporter [44] in Ad-HO-infected the 293 T-HO-CAT cells. In the Luciferase reporter, the HO target site separates the promoter from the ORF. The Luciferase expressed is short-lived, so that only sustained synthesis results in sufficient expression. New synthesis of Luciferase, measured as enzyme activity lags about an hour after DSB repair, when compared with PCR, which also showed a single prominent repaired band of correct size with no evidence of deleted products [44]. Luciferase was measured at 2 h intervals for 12 h, and the Ad-HO infected samples were compared to the uninfected samples for the same time points (Figure 3). In addition, DNA from each time point was recovered to determine the kinetics of cleavage and repair of the HO-CAT episomes. Figure 3, lower panel (CAT amplicon) shows that most of the target site is cleaved by 2 h with the repair product back to control level by 6-8 h post infection. The reporter plasmid also showed a similar pattern of Luc activity, an indication of cleavage/repair and then expression kinetics. If the HO was still active after 4 h of infection, then this would result in concomitant cutting of the Luc reporter, and be reflected as an initial delay in Luciferase expression (as evident in difference of slopes between uninfected and infected samples between 0-6 h) or slower accumulation kinetics (RLUs produced) in comparison to the uninfected control. Figure 3 shows that the pattern of Luciferase activity in the infected samples closely parallels the pattern as in the uninfected samples. Taken together, these results strongly suggest that even in a permissive system, HO gets inactivated by 4 h time point after infection, limiting its cleavage activity to just once in an experimental window of 0-12 h, or at least all the period in which ATM remains active. Of course, the situation changes once the viral replication is complete and one must take into account multiple rounds of infection in the event of absence of cell lysis. Despite the appeal of this mechanism of ATM involvement in HO inactivation, we acknowledge that our results remain correlative. A definitive proof could be obtained with the generation of a new system in AT-cells, but then again the possibility exists that it is instead ATR that is involved in phosphorylation and inactivation of HO. However, it remains clear that only one round of cutting is obtained in the first few hours of Ad-HO infection.

### High Fidelity repair of episomes via NHEJ

**Figure 4:**
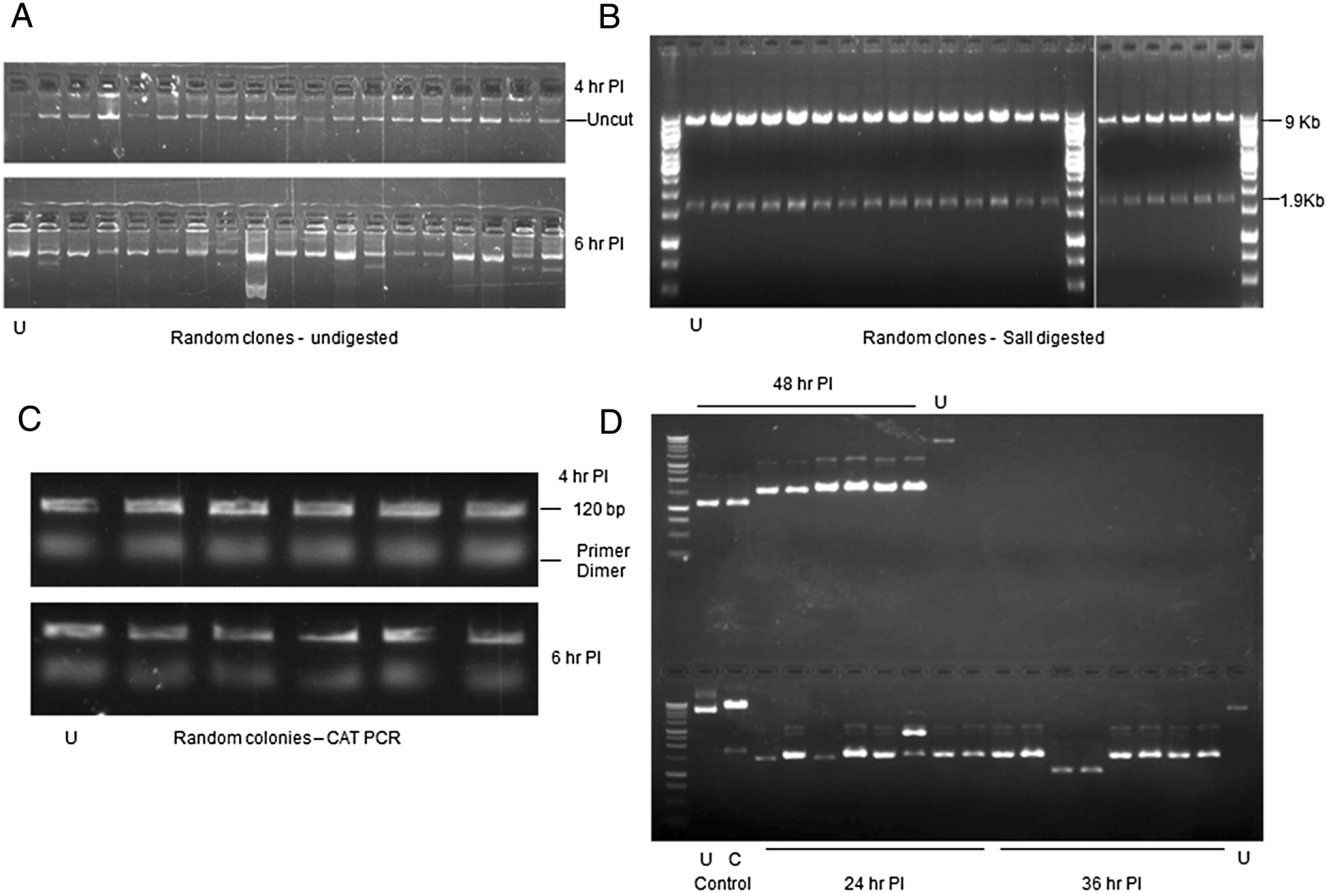
**High fidelity repair in T-HO-CAT cells. (A)** Episomes were recovered from cells after Ad-HO infection at 4 h and 6 h time point, subsequently used to transform bacteria, and plated on ampicillin plates. Boil prep of random clones from the 4 h and 6 h plates was performed to obtain the episomes and were run on 0.8% agarose gel to depict difference in size after repair (10.9 Kb in size). **(B)** SalI digestion of plasmids obtained from random clones of 4, 6, and 8 h plates PI was performed to reveal deletion in the range of 250–500 bp in size (Refer plasmid map and description in text). **(C)** PCR was performed across the DSB site using primers generating amplicon size of 120 bp with plasmids from random clones picked up from 4 h and 6 h time point plates and subsequently run in a 2.5% agarose gel to reveal micro deletions or insertions. **(D)** Accurately repaired clones were removed after isolating the episomes from cells at given time points PI by employing NheI digestion, thus enriching for inaccurately repaired plasmids (truncated ones). Episomes recovered from cells at indicated time points were digested with NheI overnight and subsequently transformed. Plasmids were subsequently obtained from random clones across different time points and were run on a 0.8% agarose gel. ‘U’ denotes uncut control plasmid. ‘C’ denotes SalI cleaved control plasmid.

We wanted to study the repair fidelity of end-joining in this episomal system. The HO site is positioned just before the chloramphenicol resistance marker (CAT) (35 bp away from the ATG start site) in the plasmid. So, any alteration in the form of deletions or insertions would profoundly affect expression of CAT and result in chloramphenicol sensitivity, detected by replica plating the LB-Ampicillin bacterial plate onto chloramphenicol Plates. 293 T-HO-CAT cells were infected with adeno-HO virus to induce the site-specific DSB. Plasmids were rescued from cells at each time point and were back transformed into supercompetent bacteria and plated on LB-Ampicillin. Somewhat to our surprise, almost all of the bacterial colonies could replica-plate on chloramphenicol and virtually all the rescued plasmids were ‘unmutated’. Boil preps were analyzed from over 200 randomly chosen clones across different time points. As shown in Figure 4A, almost all of them revealed presence of right sized plasmids (10.9 Kb). *Smith et al.,* have previously reported an average deletion size of 250 bp for a 3’ complementary overhang in a plasmid host-cell transfection assay [13]. To easily identify clones within a deletion range of 250–500 bp, we employed SalI digestion to screen random clones picked from two different time points. SalI restriction sites are present on both sides of the break site (refer plasmid map in Figure 1A) and upon digestion should give a 9 Kb fragment and a 1.9Kb fragment. We could not detect any obvious deletions among these random clones, none affecting SalI site(s), and all of them yielded correct sized fragments (Figure 4B). We also performed a PCR screen across the break using closely spaced primers (120 bp amplicon) in order to detect micro-deletions or insertions. Plasmids obtained from dozens of randomly picked colonies were subjected to PCR using these primers. However, we were unable to detect any such clones with small deletions (Figure 4C) (we previously noted that resection of a 4 bp 5′-overhang with S1 nuclease or its fill-in with Klenow was visibly shifted on a 2.5% agarose gel). In fact, most instances of rearranged clones could only be identified after digesting the rescued plasmids with NheI which has its recognition sequence on both sides and very close to the HO site. Accurately repaired plasmids would maintain the NheI site and get linearized upon digestion and thus hardly transform bacteria [The background of colonies from NheI-cut plasmids not processed for repair in mammalian cells was ~4 logs less than uncut plasmids; while for plasmids with 3′ overhangs (as produced by digesting with *Pst*I or HO) was even less]. Forced selection of plasmids (deleted in mammalian cells) in this fashion; revealed presence of extensively truncated plasmids from different time points, upon transformation into bacteria (Figure 4D).

To summarize, deletions/insertions in the recovered plasmid population from T-HO-CAT cells at different time points during the course of repair was a very rare event and could only be detected in less than 10% of rescued plasmids.

**Figure 5:**
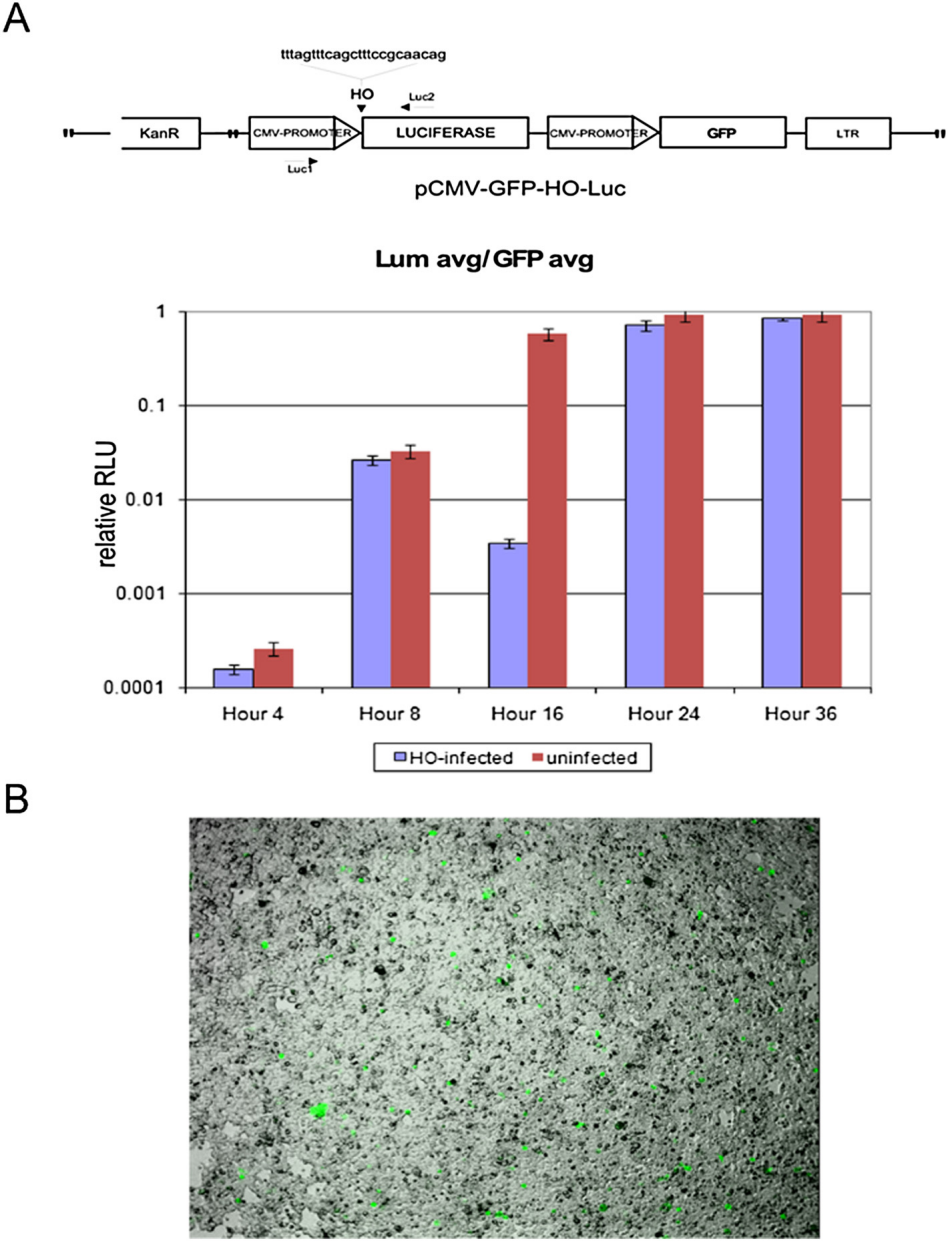
**Assay for repair fidelity in T-HO-CAT cells using luciferase reporter. (A)** Luciferase activity in 293 T cells upon transfection of HO-Luc reporter as in Figure 3 at different time points. **(B)** Demonstration of GFP-positive cells at 4 h post transfection as a measure of transfection efficiency.

As a means to quantitatively and independently estimate accurate/inaccurate repair, and also to be sure that cutting and repair was indeed taking place, we also employed the Luc reporter as in Figure 3. Since the HO site is cloned immediate upstream of the Luciferase ORF, any alteration at the repair site would result in loss of Luciferase normalized to GFP fluorescence at the undamaged site. In a transient transfection assay, 293 T cells were transfected with the reporter plasmid with or without concomitant infection with Ad-HO virus. Luciferase and GFP expression in these assays becomes clearly detectable by 4 h even with a transfection efficiency of 20-30% (Figure 5B). Maximal Luciferase accumulation (~10^6^ photons/sec - set as 1) is achieved after ~16 h (Figure 5A). An initial reduction in Luciferase light units is indicative of DSB induction due to turn over of the enzyme about 3 h later, but subsequently accurate repair reveals full recovery of luminescence (Figure 5A). These results indicated that at least in this system, NHEJ mediated end-joining is generally highly accurate.

### Metnase promotes end-processing and erroneous repair

**Figure 6:**
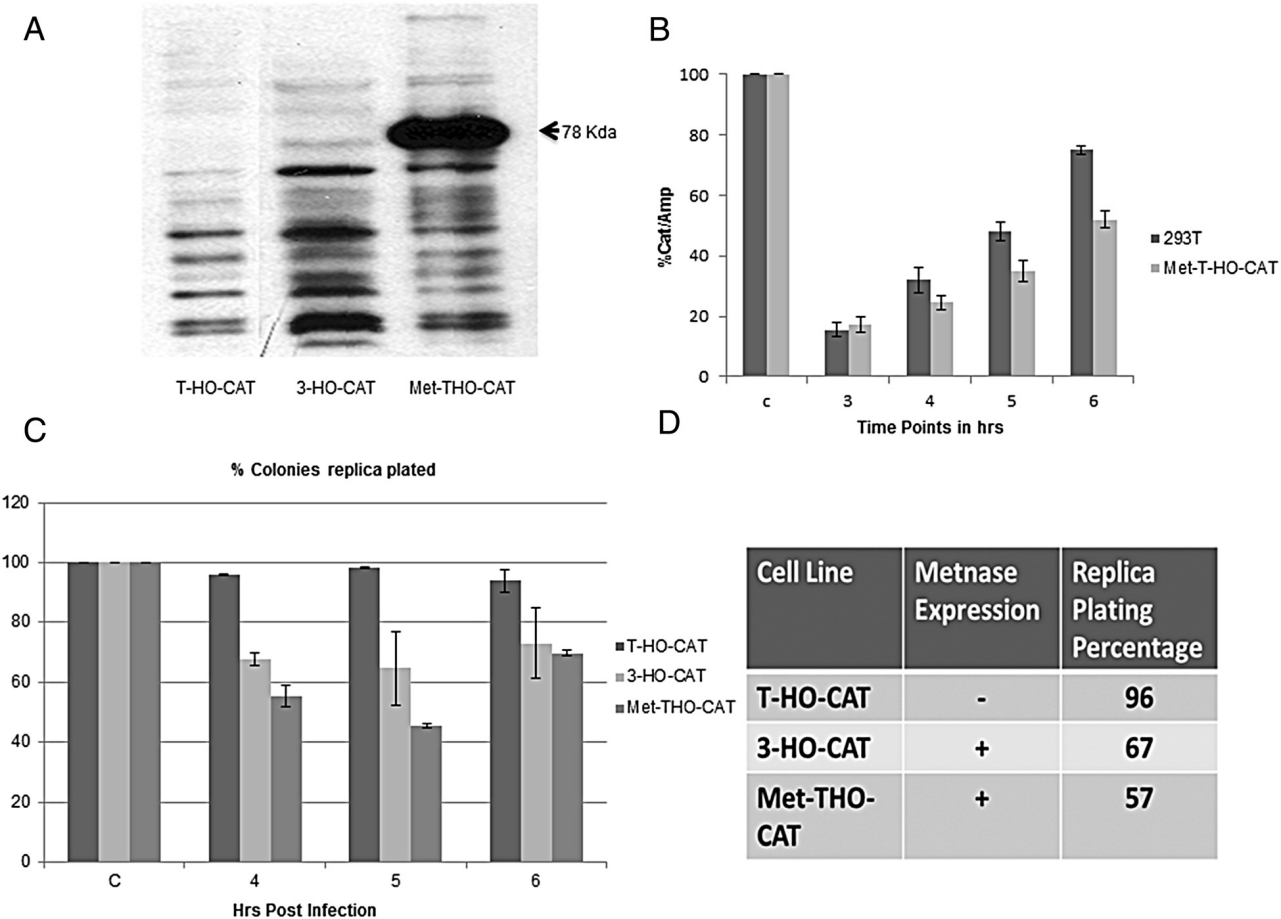
**Metnase promotes erroneous end-processing. (A)** Immunoblot showing stable Metnase overexpression in Met -THO-CAT cells along with endogenous level of Metnase in 3-HO-CAT cells. Arrow marks the correct size band for Metnase in the indicated cells. **(B)** qPCR analysis of kinetics of episomal cleavage and repair in 293 T cells (without Metnase) and Met-T-HO-CAT cells (expressing Metnase) as described in Figure 1B &1C. qPCR values are represented as (CAT/AMP)% over control. **(C)** Replica plating assay conducted for indicated time points in three different cell lines with varying Metnase expression. **(D)** Table showing average replica plating efficiency obtained from 3 different time points for each cell line and from three independent experiments.

In context of DNA repair proteins, a major difference between 293 T cells and HEK 293 cells is that Metnase is not expressed in 293 T cells but is expressed in HEK-293 cells [45]. To determine the effect of Metnase expression on episomal repair fidelity, we conducted an identical study using HEK 293 cells. Stable cell line (3-HO-CAT) was generated maintaining multiple copies of pREP4/HO-CAT plasmid. We also over-expressed Metnase in the 293 T-HO-CAT cell line that lacks endogenous Metnase (Figure 6A). DSB repair assays in all the 3 cell lines were carried out in parallel and replica plating was carried out to fish out clones with altered repair junction. As shown in Figure 6C and 6D, HEK 293 cells (express Metnase) revealed a population of plasmids (30-35%) harboring deletions. Similarly, over expression of Metnase in Met-T-HO-CAT cell line (293 T cells lack Metnase) resulted in high frequencies of deletions in 40-45% of total plasmid population (we analyzed typically at least 10 plates). In addition, there were fewer overall colonies obtained, suggesting more degradation in Metnase expressing cells. These results suggest a prominent role for Metnase in endonuclease processing at least in case of DSBs that leave a 4 bp 3′ overhang. Further, using qPCR we also found that the yield of plasmids with repaired joint, generating the CAT PCR product, was lower in cells overexpressing Metnase across different time points PI (Figure 6B).

**Figure 7:**
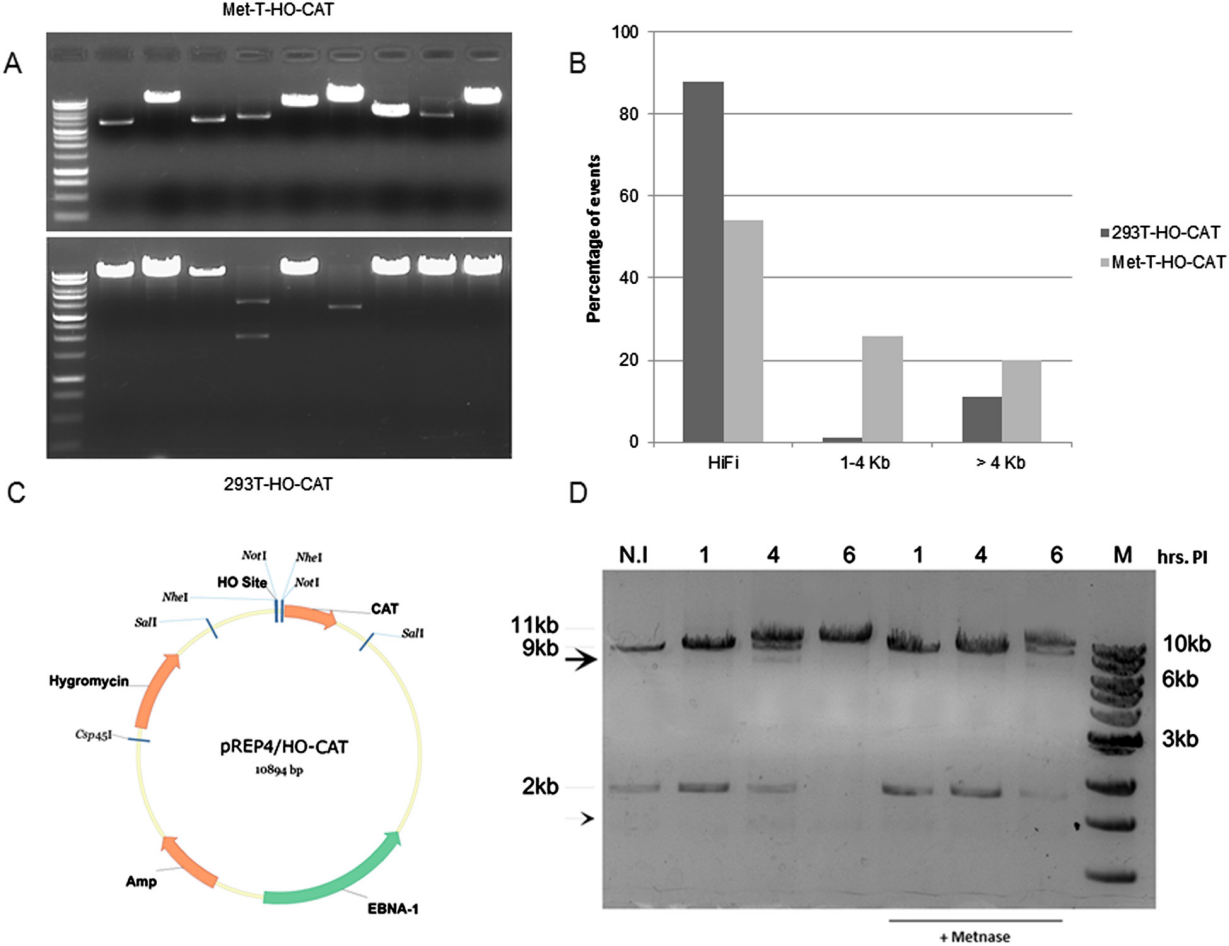
**Characterization of effect of Metnase on end-processing. (A)** Representation of deleted clones obtained from Met-T-HO-CAT cells and T-HO-CAT cells after NotI digestion of rescued plasmid population at 6 h time point post-infection. **(B)** Size distribution of deleted clones obtained from the two different cell lines. **(C)** Cartoon displaying position of different restriction enzymes on HO-CAT episome used for various digestions and southern blot. **(D)** Southern blot for pRep4-HO-CAT fragments recovered at given time points PI from cell lines in presence or absence of Metnase, after appropriate restriction digestion (see the description in text). N.I. denotes the episomes recovered from non-infected cells. Different arrow positions show processed episomal products. 11Kb size arrow denotes the singly-cut plasmid. M denotes the molecular weight marker, that is NEB 1 kb ladder labeled with PNK and γATP^32^.

Further, to assess the frequency and nature of rearrangements, we removed the accurately repaired clones from the rescued plasmid population (at 4 h PI) by NotI digestion (refer to plasmid map in Figure 7C) from both 293 T-HO-CAT cells and Met-T-HO-CAT cells. A large increase in number of colonies that did not replica-plate was obtained with episomes rescued from Met-T-HO-CAT cell line in comparison to T-HO-CAT cells, independently confirming results obtained in Figure 6C. As shown in Figure 7A (upper panel), presence of Metnase generated a population of plasmids widely varying in their deletions. A significantly smaller number of clones were recovered from T-HO-CAT cells for the corresponding time point (Figure 7A, lower panel and Figure 4D) and almost all of them are extensively deleted. Figure 7B shows distribution of random clones obtained from these two different cell lines with different extent of deletions.

### Study of early repair events and nucleolytic processing of the episomes after HO induced cleavage by Southern blotting

To probe the cleavage and repair of episomes at a molecular level, we resorted to southern blotting at different time points post-Ad-HO infection. Such a method is expected to emphasize the most prominent cleavage products obtained in the HO-infected population, to generate prototypical episomal processing patterns. The control plasmid (pREP4-HO-CAT from non-infected cells) was digested with Csp45I and NotI to yield bands of size close to 9Kb and 2Kb (N.I. 1^st^ lane, Figure 7D). Plasmids rescued at different time points from infected cells were digested with Csp45I only (refer plasmid map in Figure 7C). As the NotI site is adjacent the HO site, we should expect to observe a similar banding pattern as for the control (1^st^ lane) upon action by HO endonuclease. Figure 7D shows an expected cleavage pattern. Interestingly, we also observed a band migrating faster than the 9 Kb product (see 4 h time point, arrowhead) and a smaller species of approximately 1.5 kb that could represent further nucleolytic processing of DNA fragments following the initial HO cleavage.

In the presence of elevated Metnase expression (Met-T-HO-CAT cells), quantitatively the recovery of intact episomes after the given time window for repair (indicated as 11Kb band product) was less in comparison to episomes recovered from cells without Metnase (compare the 4 h and 6 h PI lanes; Figure 7D). Of note, we would like to remind that similar results were obtained with quantitative PCR showing that overexpression of Metnase delayed the kinetics of repair, resulting in diminished yield of repaired episomes for a given time point in comparison to control (Figure 6B), independently confirming the southern blot finding.

Sequencing of deleted clones obtained from Metnase over expressing cells revealed a recurrent pattern of DNA resection observed at the break site. Many clones lack a region of sequence, close to 200 bp in length, upstream of the cleavage site (leftward) while getting more heterogeneously resected (5′ to 3′ direction) on the other side of the DSB (Additional file 3: Supplementary text files 1 & 2). This is consistent with a very similar observation previously reported in yeast for HO induced breaks [46]. There is remarkable similarity in terms of position and segment length of the missing region found on different clones, which are of different sizes. In this regard, it is worth mentioning a very recent report by Adkins *et al.*, involving studies done with nucleosomal substrates, presented evidence for requirement of a nucleosome-free gap region adjacent to the DSB for Sgs1-Dna2 dependent resection machinery [47]. Very recently, resection proteins Sgs1 and Exo1 have been also implicated in G1 checkpoint activation in budding yeast [48]. Of note, a nucleosome plus the linker region is approximately 200 bp of DNA sequence. We also had made similar observations for precise removal of nucleosomal-length fragments at the single genomic HO-DSB in the MM3MG model when in the context of heterochromatin [17]. This suggests the involvement of a common mechanism for providing a template more amenable for endonucleolytic processing, especially active in presence of Metnase. As a last observation we should note that the Southern blots also indicate that the repair mechanism appears to be predominantly accurate NHEJ (simple plasmid re-ligation) for these episomes, as we have rarely observed evidence of formation of concatemers, even when the population of plasmids was not completely cut, and thus offering intact template strands for HR between plasmid molecules, thus presumably resulting in Holliday junctions and concatemeric units that we were unable to detect on Southern blots. Even after presumably inhibiting NHEJ with a general inhibitor of PIKs (Wortmannin) or a more specific inhibitor of DNA-PKcs (KU55933) and hence shifting the balance more in favor of HRR, the majority of repair was again NHEJ and the proportion of concatemers (based on position of the gel) was hardly increased (Additional file 4: Figure S3). The Southern blot showed that NHEJ was inhibited by the KU55933 (reduction of the 11 kb re-ligated product) but there was only a modest shift toward formation of concatemers (generated via HRR).

## Discussion

In this report, we present results from an episomal model system to study end-joining process in intact mammalian cells upon induction of a DSB. This system is designed to overcome the limitations of existing DSB repair assays and gives the opportunity to evaluate end-joining fidelity of multiple DSBs in a single cell with 4 bp 3′ overhang *in vivo.* We also evaluated the role of Metnase and its contribution towards determining repair fidelity in this system. We found that > 90% of the recovered clones from the T-HO-CAT cells, which lack endogenous Metnase, are precisely rejoined indicating high fidelity of repair (Figure 4). This is in agreement with some previous reports indicating highly accurate NHEJ in mammalian cells [49-51]. In fact, a very recent report suggested the repair accuracy to be as high as 99% in a modified I-SceI mediated cleavage and repair assay and also showed precise ligation to be mediated by classical NHEJ components [52]. However, Metnase expression may play a unique role in determining the fidelity of repair specifically in humans. Metnase is only known to be expressed in anthropoid primates (humans and apes). It has both histone methylase and DNA endonuclease activity and has been shown to promote NHEJ-mediated erroneous repair of DSBs (for a review [53]). Previous reports using cell extract based assays have been inconclusive in determining the exact role of Metnase in processing of DSBs with overhangs [29,30]. Upon Metnase overexpression, we observed a 25-30% of inaccurately repaired plasmids by replica plating assay. Molecular analysis of recovered clones and direct southern blot results independently confirm this observation. This is suggestive of a prominent role of Metnase as an endonuclease for processing of DSBs. The southern blots specifically indicate a loss of full-length repaired products (compare 6 h time point of T-HO-CAT and Met-T-HO-CAT cells in Figure 7D). This is in accordance to a recent report using cellular extract where addition of Metnase did not increase yield of repaired products but resulted in increased resection and seemed to inhibit repair [30]. However, by Southern blotting we also did not find evidence of a role for Metnase in preventing larger deletions (Figure 7B) as previously reported [27]. These findings, along with evidence of clones with precise removal of a nucleosomal-length fragment at the left (5′ side) of the break may implicate a broader role of Metnase in determining the outcome of end-joining events in mammalian cells.

## Conclusion

We have carried out a careful analysis of the fidelity of repair via NHEJ in mammalian cells using a high copy number episomal system that can be cleaved to generate homogeneous DSBs, and found that rather than error-prone, the repair was highly accurate in most cases. NHEJ was the predominant pathway of repair in this system, where the majority of plasmid was cleaved and leaving minimum number of intact copies for HR. The outcome of the accurate repair depended on presence of Metnase, a nuclease present in human cells, and which generated a pattern of discrete deletions near the DSB and resulting in much less accurate repair.

## Competing interests

The authors declare that there are none with publication of this study.

## Authors’ contributions

ADB and AR and RH conceived the study and wrote the manuscript. AR carried out the experiments. All authors have approved the final manuscript.

## Supplementary Material

Additional file 1: Figure S1 DNA damage response (DDR) activation in 293 T-HO-CAT cells upon Ad-HO infection. Infection with Ad-HO virus and generation of a single DSB in episomes (in T-HO-CAT cells) results in the phosphorylation of ATM at S1981 (activation), TLK1 (S695, inhibition), and H2AX (S139). Infection of 293 T cells that do not contain the HO-targeted episomes does not result in sufficient ATM activation (bottom panel). Cells were infected and whole cell lysates were collected at indicated time points and immunoblotted with appropriate antibody. “C” denotes the uninfected control. Drug combination (HU + TFP) was used as a positive control for ATM activation due to DNA damage (Reproduced with permission from [34]). Click here for file

Additional file 2: Figure S2 pS/T-Q status in 293 T cells upon doxorubicin treatment. WB for pS/T-Q proteins of 293Tcells incubated or not with doxorubicin and then allowed to recover after removing the drug for different hours (R1, R3, R5). Click here for file

Additional file 3 **Supplementary text files 1 and 2.** Sequencing data and alignment from selected deleted clones. Click here for file

Additional file 4: Figure S3 Evidence for presence of rare concatemers after DN-PKcs inhbition. 293 T-HO-CAT cells were treated 2 h prior to Ad-HO infection either with Wortmannin (20 μM) or KU55933 (10 μM) (potent ATM inhibitor). Episomes recovered from cells at indicated time points and were processed as described previously in text and Figure 7. Concatamers can be observed as slow migrating high molecular weight forms (> 11Kb). Click here for file
