# Supplementary figures and images for: Fidelity of end joining in mammalian episomes and the impact of Metnase on joint processing
Source: BMC Mol Biol. 2014 Mar 22;15:6. doi: 10.1186/1471-2199-15-6 (PMC3998112; doi:10.1186/1471-2199-15-6)

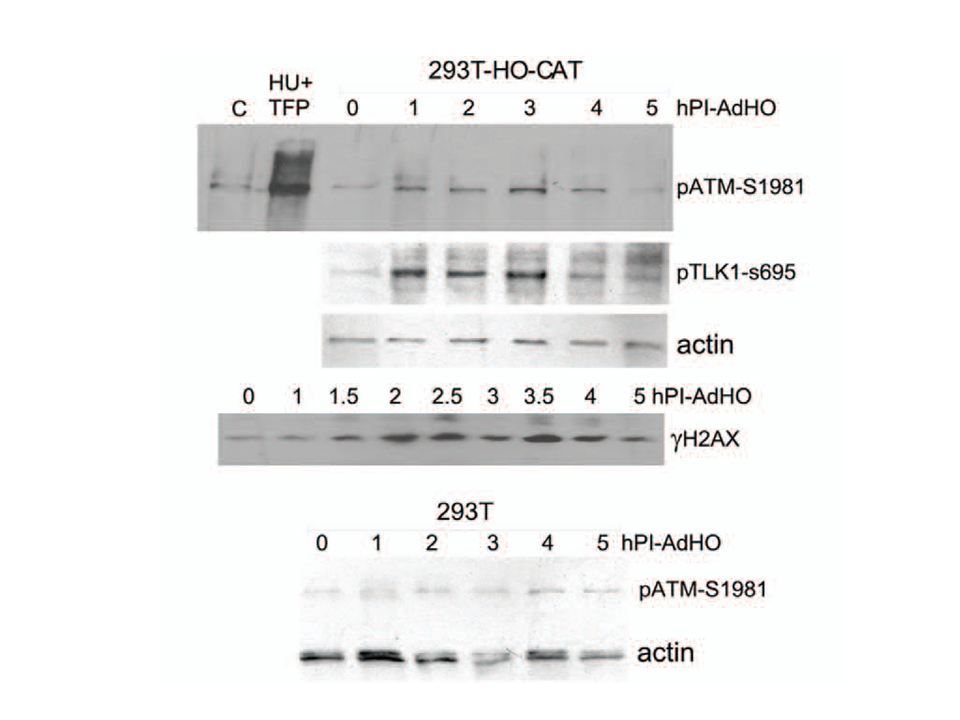

Supplement: Additional file 1: Figure S1 — DNA damage response (DDR) activation in 293 T-HO-CAT cells upon Ad-HO infection. Infection with Ad-HO virus and generation of a single DSB in episomes (in T-HO-CAT cells) results in the phosphorylation of ATM at S1981 (activation), TLK1 (S695, inhibition), and H2AX (S139). Infection of 293 T cells that do not contain the HO-targeted episomes does not result in sufficient ATM activation (bottom panel). Cells were infected and whole cell lysates were collected at indicated time points and immunoblotted with appropriate antibody. “C” denotes the uninfected control. Drug combination (HU + TFP) was used as a positive control for ATM activation due to DNA damage (Reproduced with permission from [34]). [file 1471-2199-15-6-S1.tiff]

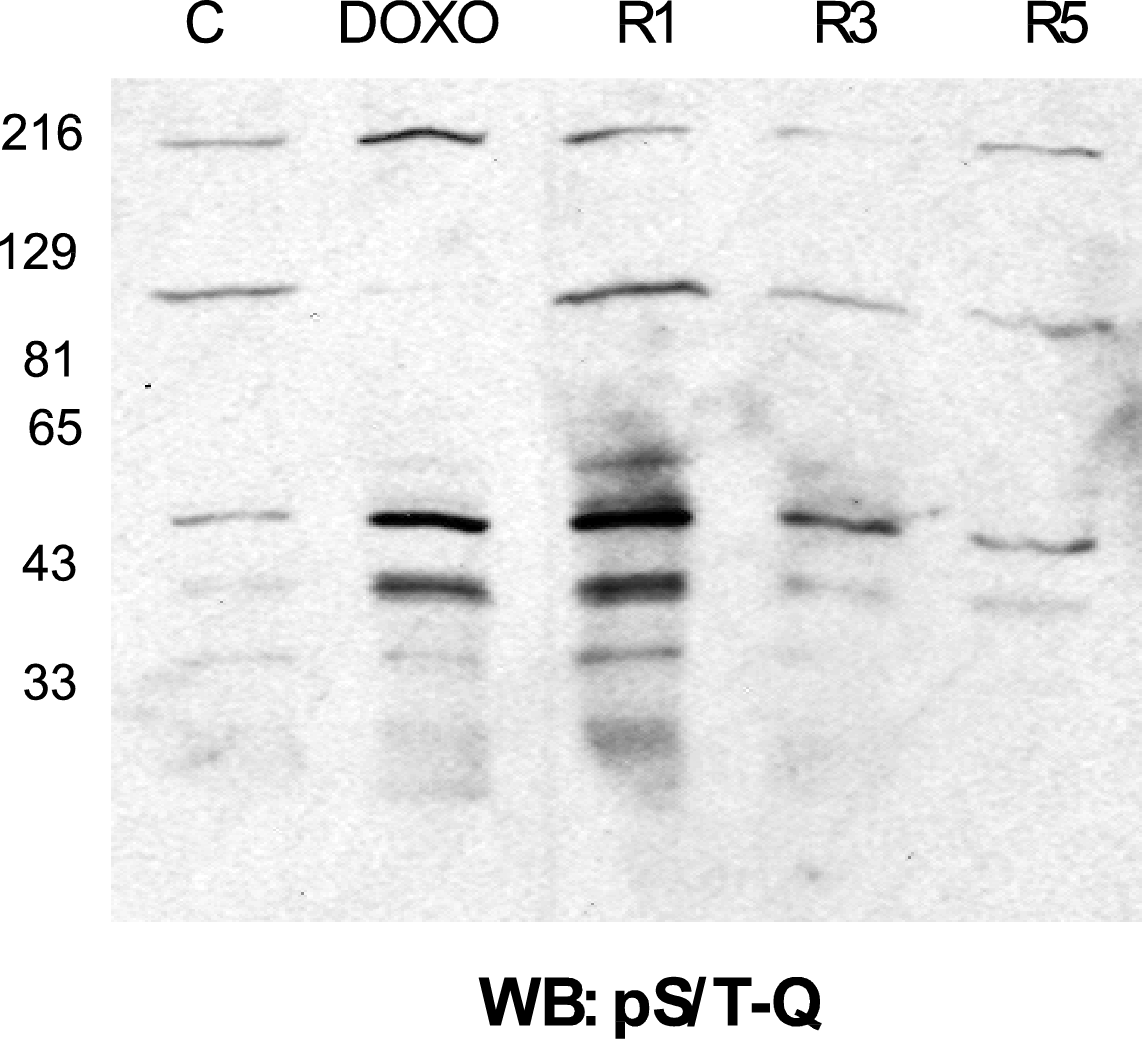

Supplement: Additional file 2: Figure S2 — pS/T-Q status in 293 T cells upon doxorubicin treatment. WB for pS/T-Q proteins of 293Tcells incubated or not with doxorubicin and then allowed to recover after removing the drug for different hours (R1, R3, R5). [file 1471-2199-15-6-S2.tiff]

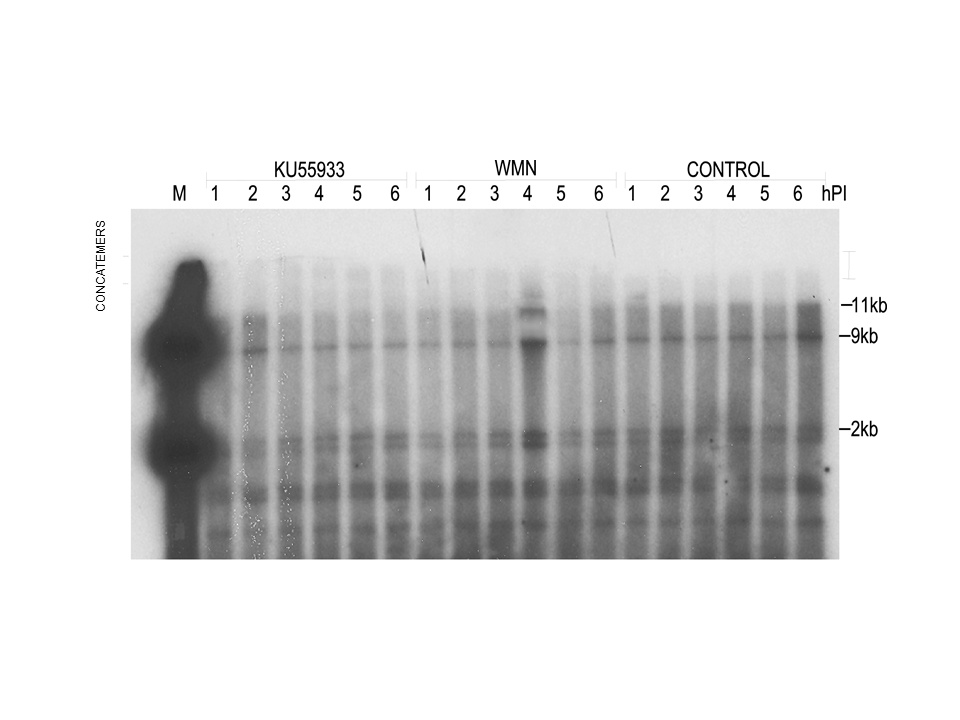

Supplement: Additional file 4: Figure S3 — Evidence for presence of rare concatemers after DN-PKcs inhbition. 293 T-HO-CAT cells were treated 2 h prior to Ad-HO infection either with Wortmannin (20 μM) or KU55933 (10 μM) (potent ATM inhibitor). Episomes recovered from cells at indicated time points and were processed as described previously in text and Figure 7. Concatamers can be observed as slow migrating high molecular weight forms (> 11Kb). [file 1471-2199-15-6-S4.tiff]
